# Deubiquitination in cancer stem cells

**DOI:** 10.18632/aging.101182

**Published:** 2017-02-12

**Authors:** Julia M. Fraile, Diana Campos-Iglesias, José M. P. Freije

**Affiliations:** Departamento de Bioquímica y Biología Molecular, Facultad de Medicina, Instituto Universitario de Oncología, Universidad de Oviedo, 33006-Oviedo, Spain

**Keywords:** degradome, ubiquitin-specific, protease, USP54, stem cells, carcinogenesis, metastasis

The hierarchical model of tumor growth postulates that the origin and progression of tumors depend on a population of cancer cells with stem properties, known as cancer stem cells (CSCs). Similar to stem cells, CSCs are able to self-renew and propagate the tumor, as well as to differentiate into a variety of cancer cell lineages, maintaining a significant degree of cellular heterogeneity within the tumors. According to this model, CSCs are responsible for tumor initiation, progression, metastasis and therapy resistance [1]. Among the multiple tumor types from which CSCs have been isolated and characterized, colorectal cancer stands out as a paradigmatic example of neoplasia in which CSCs represent the cancer cell population responsible for tumor progression and recurrence [2].

Stemness induction and maintenance involves multiple cellular pathways in whose regulation the ubiquitin-proteasome system plays a critical role [3]. Protein ubiquitination status is regulated by deubiquitinases or DUBs, a large group of proteases with the ability to hydrolyze the peptide or isopeptide bonds that link the C-terminal group of ubiquitin to the ɛ-amino group of lysine side chains of target proteins [4]. The human genome encodes at least 100 DUBs that are classified into six families according to sequence and structural similarities: ubiquitin-specific proteases (USPs), ubiquitin carboxy-terminal hydrolases (UCHs), ovarian-tumor proteases (OTUs), Machado-Joseph disease protein domain proteases (MJDs), JAMM/MPN domain-associated metallopeptidases (JAMMs) and monocyte chemotactic protein-induced protein (MCPIP) family [5]. The USPs constitute the largest family of DUBs described to date, with more than 50 members [6]. Over the last few years, numerous reports have described the implication of members of this protease family in multiple biological processes that are frequently altered in cancer. Thus, mutations and changes in the expression levels of many USPs have been associated with tumor progression [5]. However, the oncologic relevance of many USPs and their implication in CSC biology remained largely un-explored.

By analyzing transcriptional data from intestinal epithelial cells, we have recently demonstrated that *USP54*, a previously uncharacterized deubiquitinase, is overexpressed in colorectal CSCs [7]. Furthermore, we have found that *USP54* downregulation in colon cancer cells decreases their proliferation, colony formation capacity, invasiveness, and tumorigenicity when injected into immunodeficient mice, suggesting that USP54 could promote colorectal carcinoma through the regulation of stem cell properties.

The generation of gain- and loss-of-function animal models has contributed to clarify the relative importance of individual USPs in health and disease. Therefore, to further evaluate the *in vivo* role of USP54 in cancer, we generated mutant mice deficient for this deubiquitinase, which allowed us to determine that USP54 function is dispensable for normal mouse development and survival. Furthermore, the application of carcinogenesis protocols on these mice revealed that Usp54 plays an oncogenic role in azoxymethane-induced colon carcinoma. Thus, *Usp54*-mutant mice developed less adenocarcinomas than wild-type animals, with a remarkably lower incidence of infiltrating adenocarcinomas. Furthermore, these animals showed a less severe colitis when compared to control mice, supporting that Usp54 deficiency impairs the development of colorectal carcinoma. In agreement with these results, we found that *USP54* was significantly overexpressed in human adenomas compared to matched normal mucosa. Additionally, higher levels of *USP54* expression in patients with intestinal cancer were associated with lower survival, confirming the pro-tumorigenic role of USP54 in colorectal carcinoma.

To further evaluate the functional relevance of USP54 beyond colorectal carcinoma, we analyzed the effect of *Usp54* downregulation on the formation of experimental lung metastasis. With this purpose, we used B16F10 murine melanoma cells transduced with either *Usp54*-specific short-hairpin RNAs or control vector (pLKO.1) and found that *Usp54* silencing decreased the number of lung metastases. Furthermore, we explored publicly available cancer genome databases (http://cbioportal.org) and found that *USP54* is frequently mutated in pancreatic and endometrial carcinomas and melanoma, supporting a pro-tumorigenic role of USP54 also in these pathologies. Although mutations in *USP54* have been described in human acute lymphoblastic leukemia [8], our work is the first to explore the functional relevance of this proteolytic enzyme in solid tumors.

Important questions related to the oncologic relevance of ubiquitination-deubiquitination processes taking place in CSCs and their niches remain open. Besides USP54, other DUBs are likely to play important functions in CSC biology. Moreover, given the variable hierarchical organization of different neoplasias and the wide diversity of ubiquitin ligases and DUBs, it is reasonable to expect that some enzymes play pro- or anti-tumorigenic roles in different cancer types. Finally, further studies will be necessary to understand the molecular mechanisms underlying USP54 role in cancer progression and to explore the translatability of these findings into clinical benefits for cancer patients.

**Figure 1:**
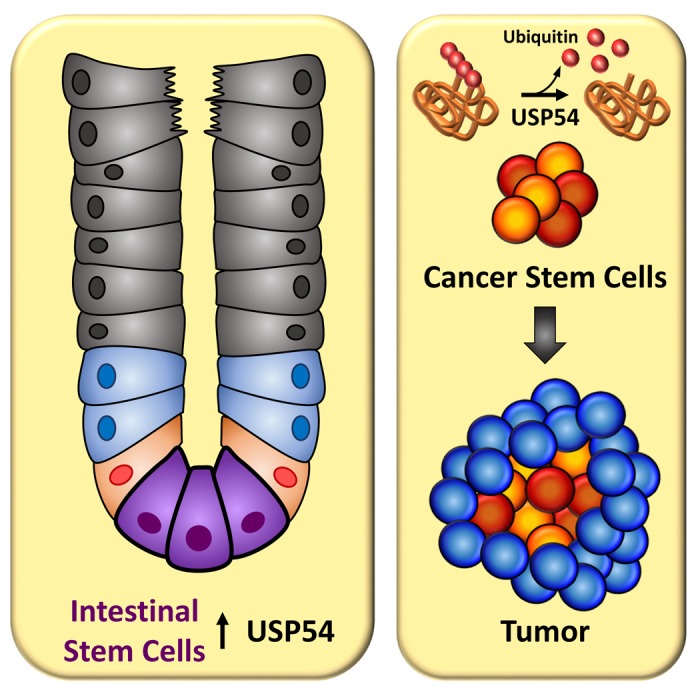
Deubiquitinases regulate stem cell biology *USP54* is overexpressed in intestinal stem cells and promotes colorectal carcinoma progression, probably through the regulation of cancer stem cell properties.
